# An Industrial Clinic

**Published:** 1921-04-30

**Authors:** 


### AN INDUSTRIAL CLINIC.

Many opinions have been voiced during the recent industrial controversy. Some have shown that there is nowadays an increasing appreciation of industrial life as it really is. Others still obviously mistake the facts.

Modern physicians have little or no familiarity with industrial processes and conditions. They have, in fact, but small interest in them. The public-health services and men practising in essentially industrial areas form necessary exceptions, but among these their relatively small numbers, circumstances, and the lack of institutional facilities reduce the possible amount of minute investigational work to a minimum. Therefore one reads with interest the records of a true industrial clinic now definitely incorporated in a large general hospital in America. Under general-hospital conditions it is possible to bring to light not only etiological factors, but in every way diagnosis of disease in the working-class patient becomes more accurate. Again, there is facility for record keeping and "following-up," without the actual treatment being impaired in the process. It is essential, if industrial health problems are to be tackled efficiently, that the workers should be cared for and registered by a department interested and competent to deal with their illness in all its social and technical ramifications.

The patient may be secondarily referred to special sections of the hospital, but the essentially industrial clinic, when in a general hospital, allows an initial grouping of cases, an observation of particular classes, and diagnostic analyses unapproachable, except at great cost, by the isolated unit. In England, specific occupational disease is, scientifically, relatively well in hand. Also, in its broad aspect, the causal relationship between industrial conditions and the commoner diseases is manifest to most of us. But of the finer details, knowledge is sadly lacking. The recently announced project of a Chair of Industrial Medicine at St. Mary's is a good step forward. It must, however, be followed by the development of true hospital industrial clinics. Thus only may the greatest questions of industrial medicine be correctly faced.

